# How We Teach and Assess Competencies in Evidence‐Based Veterinary Medicine in Preclinical Veterinary Coursework by Integrating the Expertise of a Veterinary Pharmacologist and Information Specialists/Librarians

**DOI:** 10.1111/jvp.70084

**Published:** 2026-05-06

**Authors:** Heather K. Moberly, Virginia Fajt, L. M. Rey

**Affiliations:** ^1^ University Libraries The Pennsylvania State University University Park Pennsylvania USA; ^2^ Veterinary Physiology and Pharmacology, College of Veterinary Medicine and Biomedical Sciences Texas A&M University College Station Texas USA; ^3^ Small Animal Clinical Sciences, College of Veterinary Medicine and Biomedical Sciences Texas A&M University College Station Texas USA

**Keywords:** clinical skills, competencies, EBVM, veterinary education

## Abstract

At the Texas A&M University College of Veterinary Medicine (TAMU‐CVM), the veterinary pharmacology faculty and library faculty have collaborated to teach aspects of Evidence‐Based Veterinary Medicine (EBVM) since 2010. These skills are integral to drug and therapeutic decision‐making and are required for veterinary graduate Day‐One competency. Herein, we explain the progression of incorporation of EBVM teaching at TAMU‐CVM to make clear that the development of teaching and assessment activities did not occur as a single design exercise, but rather in an iterative and reflective manner over several years. We describe the courses in which we have one or more lecture or laboratory sessions focused on scaffolding the skills of EBVM across 3 semesters, including the skills of writing clinical questions, searching the biomedical literature for evidence, critically appraising the evidence, and then applying the evidence to answer the clinical question to make a clinical recommendation. We share the specific contributions of the librarians and the pharmacologist in creating opportunities for students to develop the competencies of EBVM.

## 1 Introduction

The conceptualization of evidence‐based practice is rooted in clinical epidemiology, with the first ideas codified by David Sackett and colleagues in the mid‐1990s (Sackett et al. 1996). Soon thereafter, the seminal textbook, “Evidence‐Based Medicine: how to practice and teach EBM,” was published (Sackett 1996); this book is now in its 5th edition. Veterinary medicine began explicitly touting similar principles with the publication of Cockcroft and Holmes' book, “Handbook of Evidence Based Veterinary Medicine,” in 2003 (Cockcroft and Holmes 2003), followed by the founding of the Evidence Based Veterinary Medicine Association (Anonymous 2025) in 2006. In our work, we generally define evidence‐based veterinary medicine (EBVM) as the formal strategy to integrate the best research evidence available, the education and experience of the clinician, and the needs, wishes, and abilities of each client to make clinical decisions. In short, this strategy starts with asking a specific clinically‐relevant question, searching for evidence about the question, appraising the evidence, and then applying it to the question to make a recommendation in a clinical setting.

Although teaching evidence‐based medicine principles has long been incorporated into medical education (Ahmadi et al. 2015; Ilic and Maloney 2014; Kumaravel et al. 2020; Rodriguez et al. 2023; Simons et al. 2019), there is more variability in veterinary education. Teaching tutorials, like the RCVS Knowledge EBVM Learning tutorials (2015, 2020, and 2022) (RCVS Knowledge 2025), were developed for use in and beyond the curriculum. The most recent study on the formal incorporation of EBVM principles and skills into veterinary education curricula suggests inconsistencies across veterinary schools (Shurtz et al. 2017). One likely reason for inconsistencies is the lack of agreement about what the most important skills are and the best approaches to incorporate them into veterinary training. For example, at some institutions, there are stand‐alone courses called EBVM, whereas at others, EBVM is incorporated into epidemiology and public health courses. More important than the names of courses or the location of the content is agreement on what the new veterinarian is expected to know, or know how to do, in the context of EBVM.

The American Association of Veterinary Medical Colleges Competency‐Based Veterinary Education (AAVMC CBVE) Working Group has developed competency frameworks applicable across many models and settings of veterinary curricula (AAVMC Council on Outcomes‐based Veterinary Education et al. 2024). The CBVE Framework and the CBVE Entrustable Professional Activities provide high‐level outcomes for new graduates of veterinary professional programs (American Association of Veterinary Medical Colleges 2025). These program level competencies are meant to be achieved through the development of subcompetencies, and pharmacology‐related subcompetencies have been mapped to the CBVE framework to enable pharmacology educators to create effective educational activities and content (Hawes et al. 2024; Werners and Fajt 2021). Table 1 illustrates the consensus Day‐One basic and clinical pharmacology competencies mapped to the program‐level outcomes relevant to EBVM.

**TABLE 1:** Mapping of day‐one pharmacology competencies about EBVM to program level competencies from the AAVMC CBVE.

AAVMC CBVE domain	AAVMC CBVE competency	Mapped Day‐One pharmacology competency (Hawes et al. 2024; Werners and Fajt 2021)
Domain 7	Competency 7.4—Engages in self‐directed learning	List the steps of evidence‐based medicine and the hierarchy of evidence as it relates to pharmacologic therapeutic interventions and interpret and apply evidence to a clinical patient. Describe the relative bias associated with various study designs in pharmacology and therapeutics, including clinical trials, pharmacokinetic studies, narrative and systematic reviews, case reports, and in vitro studies. Compare and contrast different sources of drug information and appropriately apply that information to individual patients/populations. Integrate new drug information into existing personal and clinical practice knowledge base, and then incorporate the information into clinical practice and decision‐making
Domain 9	Competency 9.1—Practices evidence‐based veterinary medicine	
Domain 9	Competency 9.2—Disseminates knowledge and practices to stakeholders	

At the Texas A&M University College of Veterinary Medicine (TAMU‐CVM), the veterinary pharmacology faculty and library faculty have collaborated to teach aspects of EBVM since 2010. This collaboration is in keeping with the work of the academic division of the American Library Association, the Association of College and Research Libraries, which published a Framework for Information Literacy for Higher Education (Framework) (American Library Association 2015). This Framework calls for greater collaboration between faculty (meaning subject teaching faculty) and librarians (who are faculty at some institutions) to address core information concepts in the higher education context. Because librarian collaborations have been reported in other health professions but not in veterinary education (Nicholson et al. 2024, 2020; Cyrus et al. 2025; Gill and Parker 2022), herein we report on the collaboration of a pharmacologist and librarians at our institution. We describe the progression of incorporation of EBVM teaching at TAMU, so other programs might adapt and follow the approach. We chose to describe the evolution of our teaching about EBVM because we want to make it clear that we did not design the learning activities and insert the contact hours into the curriculum as a single design exercise, but rather in an iterative and reflective manner over several years. In addition, it should be noted that we describe only what the authors teach at our institution. We know that other faculty provide opportunities for developing similar or related skills, such as requiring analysis of scientific literature in other core or elective courses, but they are not all mapped in our curriculum, and to our knowledge, they do not include librarian expertise and collaboration.

## 2 Our Context and How Our EBVM Collaboration Developed

The veterinary program at TAMU is a 4‐year professional program leading to a DVM (Doctor of Veterinary Medicine), which is preceded by 2 or more years of undergraduate prerequisite coursework including biology, chemistry, statistics, and communications. The collaborators on this endeavor include a pharmacologist (V.F.) and two librarians (L.M.R. and H.K.M.). V.F. has taught pharmacology in the second year of the DVM program since 2005; both librarians were previously affiliated with the TAMU Medical Sciences Library, and L.M.R. is now a faculty member in Small Animal Clinical Sciences. V.F. and H.K.M. were early members of the Evidence Based Veterinary Medicine Association and believed that incorporating EBVM principles into foundational courses would demonstrate its value to clinical practice. In addition, V.F. and H.K.M. have served on the DVM Curriculum Committee, providing opportunities to shape program‐level competencies in EBVM.

When V.F. taught EBVM concepts in pharmacology in 2006–2010, no librarians were included. In addition, there were no TAMU‐CVM program level outcomes identified specifically as EBVM, although there were program‐level competencies somewhat relevant to EBVM in that students were required to log the reading of 20–40 peer‐reviewed papers sometime during the 4 years of the program.

As members of the DVM Curriculum Committee, in 2013, the pharmacologist (V.F.) and one librarian (H.K.M.) facilitated a revision of program level competencies in the domain of Critical Analysis of New Information and Research Findings Relevant to Veterinary Medicine. The edited competencies, which each had to be demonstrated five times, became:

- Apply evidence from peer‐reviewed literature to a clinical case.
- Create a PICO‐based clinical question that addresses a knowledge gap.
- Critically appraise published references.
- Search CAB Abstracts and identify potentially useful references to address a knowledge gap.
- Search PubMed and identify potentially useful references to address a knowledge gap.

As the program level outcomes were changing, from 2014 to 2018, the time and involvement of the librarian increased from leading one session to attending all EBVM‐related sessions in the pharmacology courses to model the need for collaboration when acquiring these skills (see Table 2). Over several years, we modified the checklists and rubrics used in class (see Table 2), and we eventually deposited them in OAKTrust, TAMU's online institutional repository, giving them Creative Commons licenses Attribution‐NonCommercial‐ShareAlike 4.0 International Licenses (CC‐BY‐NC‐SA), which allows others to use and modify them (Fajt and Moberly 2022) Reflecting together and alone, we arrived at similar conclusions; the uneven nature of student experience and prior instruction related to searching for literature created uneven skill levels among students. We speculated that students often model faculty and veterinary practitioners and observed shortened processes based on subconscious competence. For example, an experienced practitioner may go directly to a resource such as PubMed and enter search terms they have had success with previously. The student does not witness the other resources, nor search terms that were tried for a search on this topic previously. The student likely will not witness the nuance of trying several resources and search term combinations, examining the types of results, and filing in memory what resources will be found where. The student likely does not know the background information known by the practitioner that provides a foundation for selecting from a results set and evaluating individual results. All this must be taught and practiced so that when the student reaches the stage where they make clinical decisions, they also have a subconscious competence and an idea about their background information related to different topics.

**TABLE 2:** Timeline of incorporation of the skills and knowledge relevant to EBVM competencies into Pharmacology courses in the DVM program at TAMU‐CVM from 2006 to 2025.

Academic year(s)	Pharmacology I	Librarian included	Pharmacology II	Librarian included
2006–2007	Lecture: the steps of EBVM		Lab: write PICO question, find some papers, ID study design and assign level of evidence, categorize the totality of evidence (participation grade only; no rubric)	
2007–2008	Lab: With a focus on sources for drug information, there is one activity on the lab worksheet to find an article to support a drug choice using PubMed (participation grade only; no rubric)		Lab: critical appraisal exercise in which papers were supplied, along with a table with a series of questions about each section of a paper [based on http://leml.asu.edu/Wu_Website_4_Students/Reading‐guides/McNeal‐How2ReadSciPaper.pdf] (participation grade only; no rubric)	
2008–2009	Same as previous year		Lecture: the steps of EBVM at end of the course	
2009–2010	Same as previous year		Lab: write a PICO question, develop list of keywords for searching, select articles to answer the question	
2010–2011	Course assignment: develop therapeutic plan and include citations of literature with level of evidence (decision‐making assignment)—LIBRARIANS join for first time in a lab session about comparing drug resources	X	Lab: write PICO questions, come up with keywords that could be used in CAB Abstracts. Lecture: guest lecturers delivered subject‐specific content, and there were exercises after 3 lectures with PICO question writing, keyword development, searches (with guiding questions to aid in performing searches), and use of abstracts to answer the PICO questions	
2011–2012	Lab: one question in the lab exercise that required searching CAB Abstracts about a specific therapeutic question		Course assignment: develop therapeutic plan and include citations of literature with level of evidence (decision‐making assignment). Lab: papers were provided; first iteration of literature evaluation form used for critical appraisal (adapted from 2011 Arlt article)	
2012–2013	None		Course assignment and lab: same as previous year. Librarians guided searching activities in lab	X
2013–2014	None		Course assignment: same as previous year; revised labs to be 3–5 lab sessions explicitly assigned to the steps of EBVM, with assignments for each step (PICO questions, search strategy, appraisals); at least the first two (asking question, searching) including librarians	X
2014–2018	None		Same as previous years	X
2018–2019	4 sessions in Public Health, 1 h in Pharm, 2 PICOs, 3 appraisals, 1 apply, Added Fact Box to Apply step—practice articles in class	X	Course removed from curriculum	
2019–2025	Same as previous year	X		

*Note:* Labs are 2 h of contact time, and lectures are 1 h.

A curriculum update was implemented for the program in 2017, which was based on “New Graduate Outcomes” (NGOs), program‐level expectations for every new graduate from our institution. These outcomes reflect fairly well the domains and competencies of the CBVE framework. Both the TAMU NGOs and the CBVE framework include outcomes related to the practice of EBVM. As a consequence of the implementation of the updated DVM curriculum, a 6‐semester series of Professional and Clinical Skills (PCS I‐VI) courses were added. These courses have 3 streams within each semester: professional skills (e.g., communication and ethics), clinical skills (e.g., physical examination and surgical skills), and critical thinking. From 2017 to 2020, an introduction to EBVM was presented by a clinician (but not including a librarian) in the 1st semester PCS course with a focus on critical reading that is, the skills associated with critical appraisal step of EBVM. Beginning in 2018, in PCS I, a librarian participated in 2nd semester stream of activities that included class sessions, group meetings with clinical faculty, and assignments, which had the learning goals of identifying and using appropriate information to answer questions about clinical problems and communicating information to professional and lay audiences. The assignments and required elements included development of a client brochure, development of a document summarizing the medical problem geared to veterinary colleagues, group meetings with clinical faculty to discuss the assigned medical issue, and group meetings with a librarian to discuss searching.

In PCS II, in 2021, with the legacy curriculum completed, the pharmacologist was invited to take over as lead of this stream of activities. Over the next 4 years, the pharmacologist and library faculty made multiple changes to what occurred in classroom sessions, what the assignments looked like, and what the focus was on. These changes were made to emphasize the EBVM skills of searching and critical appraisal. To accomplish this, the client brochure was eliminated, as were the group meetings with faculty, which were cumbersome to manage and which had focused on the content of the brochures rather than the EBVM‐related skills. The two classroom sessions, with small group meetings with the librarian between them, became focused on the skills associated with searching the biomedical literature using PubMed and CAB databases. New assignment rubrics were developed so that the searches themselves would be assessed by the librarian. In 2023 L.M.R. took over the librarian role, having transitioned from library faculty to TAMU‐CVM faculty. Further changes included more explicit instruction on the building blocks of searching (i.e., identify key concepts, develop search terms, develop synonyms for the search terms, build the search using appropriate Boolean terms, troubleshoot the search).

Prior to, and then parallel to, the evolution in the PCS courses, the pharmacologist tried a number of approaches to incorporating EBVM skills development into the two semesters (2005–2018) or one semester (2018–2025) of pharmacology (see Table 2 for details). Once the pharmacology course content was condensed from 8 credit hours over 2 semesters to 3 credit hours in one semester, with 5 contact hours in the Public Health course, classroom activities and assessments had to become streamlined, which allowed for more reflection on the critical components of the activities.

## 3 Current Approach to EBVM Collaboration

Based on the collaborative efforts of the pharmacologist and the librarians and the evolution described above, we currently address EBVM competencies in the following ways (see also Table 3): In the first year of the curriculum, in PCS I, we introduce EBVM and critical appraisal as a critical thinking concept, and in PCS II we focus more on the skills of searching for evidence in two separate lab sessions using prewritten clinical questions in the format of PICO that is, Patient/Population + Problem, Intervention, Comparison, and Outcome. In the first semester of the second year of the curriculum, the EBVM content is split between two courses: a pharmacology course and a course called “Public Health, Epidemiology and Evidence‐Based Medicine.” In this semester, each student creates their own PICO question, searches for, selects, and appraises evidence to answer their question, and makes a clinical recommendation.

**TABLE 3:** Current approach to scaffolding and progression of EBVM competencies using librarian and pharmacologist expertise in the DVM program at TAMU‐CVM.

Year/semester in the curriculum	Course	Contact hours	General objectives of the session	Assessments used^a^
1st year, 1st semester	Professional and Clinical Skills I	2	Introduce the general concepts of EBVM, provide a brief introduction to critical appraisal in which students read an assigned article in a group and review salient quality criteria	In‐class ungraded activity with check boxes for quality criteria
1st year, 2nd semester	Professional and Clinical Skills II	2	Introduce search skills using PubMed and a CABI product	Assignment in which students provide screenshots of their best searches in the two databases using provided PICO questions
1st year, 2nd semester	Professional and Clinical Skills II	2	Continue to build on and advance search skills, reinforce critical appraisal skills	Assignment in which students provide screenshots of their best searches using assigned PICO question, and a separate assignment in which students work in groups to select a paper and perform a brief critical appraisal using a provided form
2nd year, 1st semester	Public Health^b^	1	Introduce writing PICO questions	Assignment (with points attributed to the Pharmacology course) in which students write two PICO questions
2nd year, 1st semester	Public Health	1	Search workshop for a student‐selected PICO question	
2nd year, 1st semester	Public Health	1	Introduce critical appraisal in the context of evidence about pharmacotherapeutics and practice using the form for appraising the most common types of studies	Assignment (with points attributed to the Pharmacology course) in which students turn in the three best articles to answer their PICO question along with a critical appraisal of each using a provided form
2nd year, 1st semester	Public Health	1	Introduce the step of applying the evidence to PICO questions	
2nd year, 1st semester	Public Health	1	Continue working on the step of applying the evidence	Assignment (with points attributed to the Pharmacology course) in which students use the 3 articles previously appraised to answer their clinical question, make a clinical recommendation, and provide the strength of that recommendation

*Note:* Labs are 2 h of contact time, and lectures are 1 h.

^a^ Templates and descriptions of all the assessments described here are available with a CC‐BY Creative Commons License at https://hdl.handle.net/1969.1/196029.

^b^ The full name of this course is Public Health, Epidemiology, and Evidence‐Based Medicine. The contact hours related to EBVM were originally included in the Pharmacology courses, which included two semesters and more contact hours. When the curriculum was updated in 2017, Pharmacology was reduced to 3 semester hours, and the EBVM content was condensed and incorporated in the Public Health course.

## 4 Reflections on the Evolution of EBVM Teaching Activities

### 4.1 The Collaboration

From the collaborative perspective, working together on the EBVM content strengthened each of our skillsets. We recognize that when we first started collaborating, we didn't write down our reflections or take extensive notes on our experiences; our memory is incomplete and perhaps faulty. So much of what went well can be credited to conversations that advanced each person's knowledge and skills. It was a deliberate effort to work toward positive successful EBVM‐related activities.

In retrospect, one of the librarians (H.K.M.) identified distinct areas where she needed to keep current or develop skills. Although librarians keep current with information resource choices and changes in search interfaces, this needed to be at a different, higher level than teaching more generalized searching sessions. She needed to evaluate which resources and search features aligned with the competencies. The choice to require PubMed instead of a different MEDLINE interface and the CABI Publishing interface for CAB Abstracts was deliberate and differs from some other universities. She needed to read veterinary education and accreditation literature and keep up with general medical education trends and chose to earn a postgraduate certificate. She needed to take advantage of meetings with students, alumni, and practicing veterinarians and to ask questions about what these groups were searching for, where and how, and importantly *why* they were searching. Knowing the *why* allowed her to work with the pharmacologist to contextualize the searching sessions, student meetings, assignments, and grading.

One noted strength of the collaboration during each in‐person laboratory session was that the pharmacologist would share with students that she searches the biomedical literature nearly daily and yet continued to learn every year in each of the librarian's sessions and from conversations. It is our perception that students believe that either learning skills such as searching the literature is a skill they already possess and only requires a short learning session to be prepared for the rest of their career or they believe it is an unlearnable task and internet search engines will suffice. The truth is that these information literacy skills are threshold concepts for EBVM (Townsend et al. 2011; Anonymous 2005). They need to be learned, practiced, and updated regularly as platforms and technologies change, which the contextual comment from the pharmacologist supports.

### 4.2 Pharmacologist's Observations

The skills of EBVM are critical to the practice of veterinary medicine, and the learning activities and assessment described in this article demonstrate that EBVM can be incorporated into discipline‐specific teaching, rather than relegated to stand‐alone courses. In addition, V.F. observed that weaving these skills into pharmacology coursework has the potential to demonstrate to students that the skills are integral to drug and therapeutic decision‐making for clinical patients. Our experiences in the sessions in the Public Health course, which are workshops rather than didactic in nature, corroborate the findings of instructors in a medical school, who demonstrated the value of in‐person activities for developing the skills of EBVM (Magro et al. 2025).

In the context of working with librarians to develop teaching and assessment activities, it is much harder to avoid reflecting on how well activities are working when you and your colleague are planning, grading, or managing problems in real time. Painful questions from students led to many refinements over the years, and they required us to be very clear with ourselves and then with students about the clinical value of learning these skills.

### 4.3 Librarians' Observations

Students were sometimes frustrated with the searching sessions, assignments, and grading. We wondered if students were reacting to the ways the skills and assignments were presented and phrased. When we direct students to “answer the question,” are we setting them up with an unrealistic task because of the way we have phrased our direction to them? Throughout education, “answer the question” means that the question has a single answer. We found in assignments that students sought a single quantifiable answer and would seek an article or a few articles that supported a single answer, such as a treatment or a dose. Instead, our objective is for students to locate a body of evidence from which to synthesize possibilities and explore the strength of the possibilities. By phrasing the assignment, “answer the question,” did we use a shorthand used among experts with competence who understand the nuance? Could the students be more successful if we rephrase the task? Similarly, when we ask learners to “phrase your topic as a question,” do we explicitly share that the question must be answerable? There are more unanswerable questions than answerable questions.

We ask students to follow a specific path or series of steps when they search. The path begins with determining a question and, perhaps following a framework like PICO, breaking the question into concepts. They determine search terms for each concept and perform the search. When we ask students to execute this series of tasks, several things commonly happen. One is that, probably because of the ubiquity of internet search engines and single boxes into which the search is entered, the student wants to take the search terms and concepts and reform them into a prose language search. Commonly, they type the question, as a sentence, into whichever resource they have been assigned, seen used by a practitioner or student above their rank, or with which they have previously had success. Therefore, it can be difficult to persuade them to use concepts and Boolean (AND/OR/NOT) construction. When persuaded, the students either wanted few, specific results to form a succinct answer or enormous results sets, similar to an internet search engine query, from which to scroll and pick and locate specific results to, again, form a succinct answer. In assignments, students focused on the “answer” wanting to know the correct search, correct results list, and correct article or articles. Even as the teaching sessions and assignments have evolved, L.M.R. notes that first‐year students in particular continue to find searching in this way and EBVM both ambiguous and uncomfortable.

In addition to wanting phrasing to be easier, students want to only rely on articles containing all of the search concepts instead of accepting that often several articles each contribute a piece to the recommendation. Because of this, we deliberately changed what we said to the students. We now say, “you are not looking for an article or two to answer your clinical scenario.” This is not a hunt for a magic article to answer the question and if you persevere and look at enough results, you will find it. We said, “You are looking for a group of articles, which should provide options for your answer.” Although this still frustrates some students who want the grading of the assignment to tell them the correct search, the correct number of results, and the correct article(s) to answer the question, it has helped us frame our sessions in class and in conversations with students.

## 5 Future Areas of Investigation

We have anecdotal information that other courses and clinical rotations in our program address one or more of the core skills of EBVM (e.g., see Fajt et al. 2025), but there is no comprehensive map. Therefore, we would like to identify and map other learning activities in the curriculum that promote the skills of EBVM, such as searching and critical appraisal. This could reveal overlap that could be better utilized for reinforcement, as well as gaps that could be filled in. This would lead to greater alignment of the goals and a more integrated progression of skill development.

On the assessment side, in the TAMU‐CVM curriculum, there are Objective Structured Clinical Examinations (OSCEs) throughout the preclinical years. Because the EBVM content in the PCS courses is working well from our perspective, and the progression of assignments in the Pharmacology course adds credibility to the clinical nature of the skills, we would like to explore the possibility of including these skills in OSCEs. Although OSCEs are being used in medical education to assess EBM skills, there are no known reports of the use of veterinary OSCEs to assess EBVM skills (Aranda et al. 2019; Kumaravel et al. 2021). Therefore, developing an OSCE to assess searching skills is an aspirational future step.

## 6 Conclusions

We have described the strong collaboration between librarians and a pharmacologist to develop learning activities over several years to address pharmacology Day‐One competencies related to EBVM. We found that working with experts completely outside our own discipline provided a welcome perspective on what and how we teach, and even on what our habits of mind are. These different points of view enhanced the design of our teaching materials and assessments. Because of this collaboration and its duration, we are able to scaffold instruction, hold students responsible for information learned in one semester in the next, and work toward program level outcomes. In addition, the collaboration between subject‐experts such as pharmacologists and librarians/information specialists can demonstrate to students that veterinary subject specialists must learn competence with information skills along with the other skills needed for a career‐long attention to evidence‐based practice. We propose that pharmacologists at other institutions use this model to incorporate EBVM‐related pharmacology competencies with librarian collaborators to enhance their teaching and address important Day‐One competencies. We also propose that other veterinary disciplines use this approach to incorporating EBVM into those subjects without having to create new courses or even to add hours to a curriculum to enhance the instruction of EBVM principles and skills.

## Author Contributions

H.K.M. and V.F. contributed equally to conception, drafting, and review of this article. L.M.R. contributed to drafting and review of this article.

## Funding

The authors have nothing to report.

## Ethics Statement

The authors have nothing to report.

## Conflicts of Interest

Heather K. Moberly is a member of the CABI Publishing North American Library Advisory Board.

## Data Availability

Data sharing is not applicable to this article as no datasets were generated or analyzed during the current study.
